# Cigarette smoke regulates the expression of TLR4 and IL-8 production by human macrophages

**DOI:** 10.1186/1476-9255-6-12

**Published:** 2009-05-01

**Authors:** Hadi Sarir, Esmaeil Mortaz, Khalil Karimi, Aletta D Kraneveld, Irfan Rahman, Eric Caldenhoven, Frans P Nijkamp, Gert Folkerts

**Affiliations:** 1 Division of Pharmacology and Pathophysiology, Departement of Pharmaceutical Sciences, Faculty of Sciences, Utrecht University, the Netherlands; 2 Department of Animal Science, Birjand University, Iran; 3 Department of Clinical Biochemistry, Faculty of Medical Sciences, Tarbiat Modarres University, Tehran, Iran; 4 Department of Basic Science, Section of Biochemistry, Faculty of Veterinary Medicine, Urmia University, Iran; 5 Department of Pathology and Molecular Medicine, Centre for Gene Therapeutics, McMaster University, Ontario, Canada; 6 Department of Environmental Medicine, Division of Lung Biology and Disease, University of Rochester Medical Center, USA; 7 Danone Research Centre for Specialised Nutrition, Wageningen, the Netherlands

## Abstract

**Background:**

Toll-like receptors (TLRs) are present on monocytes and alveolar macrophages that form the first line of defense against inhaled particles. The importance of those cells in the pathophysiology of chronic obstructive pulmonary disease (COPD) has well been documented. Cigarette smoke contains high concentration of oxidants which can stimulate immune cells to produce reactive oxygen species, cytokines and chemokines.

**Methods:**

In this study, we evaluated the effects of cigarette smoke medium (CSM) on TLR4 expression and interleukin (IL)-8 production by human macrophages investigating the involvement of ROS.

**Results and Discussion:**

TLR4 surface expression was downregulated on short term exposure (1 h) of CSM. The downregulation could be explained by internalization of the TLR4 and the upregulation by an increase in TLR4 mRNA. IL-8 mRNA and protein were also increased by CSM. CSM stimulation increased intracellular ROS-production and decreased glutathione (GSH) levels. The modulation of TLR4 mRNA and surface receptors expression, IRAK activation, IκB-α degradation, IL-8 mRNA and protein, GSH depletion and ROS production were all prevented by antioxidants such as N-acetyl-L-cysteine (NAC).

**Conclusion:**

TLR4 may be involved in the pathogenesis of lung emphysema and oxidative stress and seems to be a crucial contributor in lung inflammation.

## Introduction

Macrophages play a central role in both specific and nonspecific immunity against bacterial, viral, and fungal infections. The unique localization of alveolar macrophages in the alveoli (between air and lung tissue) [1], represent them as the first line of defense against inhaled microorganisms or particles [2]. The role of these cells in the pathophysiology of chronic obstructive pulmonary disease (COPD) has been well documented [3,4]. Cigarette smoke (CS) stimulates various immune cells to increase the production of cytokines and generate of reactive oxygen species [1]. CS causes lung damage by oxidative stress either by itself or due to oxidants released by inflammatory cells that are recruited as a result of smoke-induced injury. CS is a major source of oxidants/free radicals and a complex of over 4700 chemical compounds [5]. This huge amount of oxidants from CS and those formed endogenously cause an imbalance between oxidants and antioxidants which are considered to be important in the pathogenesis of COPD [6,7]. Multiple intracellular signaling events occur by CS, which ultimately leads to the synthesis and release of pro-inflammatory mediators, such as interlukine-8 (IL-8), IL-1β, and tumor necrosis factor-α (TNF-α) [8,9].

The function of the innate immune system is the discrimination of invading pathogens and self-cells by utilizing signals from the Toll-like receptors (TLRs). TLRs recognize specific patterns of microbial components [10] and signals to initiate a range of host defense mechanisms [11]. TLR4 is a crucial component of the signaling receptor complex which is involved in recognition of a major integral glycolipid component of the outer membrane of gram-negative bacteria (lipopolysaccharide or LPS) [12]. Downstream signaling of TLR4 pathway includes myeloid differentiation factor 88 (MyD88), IL-1 receptor associated kinases (IRAKs), and TNF receptor-activated factor 6 (TRAF6). TRAF6 activates various kinases, which leads to I-κB degradation and NF-κB activation. Activated NF-κB translocates into the nucleus and increases the production of pro-inflammatory mediators like IL-8 [13-15]. The redox status of cells contributes to the modulation of NF-κB. Moreover, ROS regulate immune-inflammatory cellular signaling via TLR4 by activation of NF-κB [16,17]. Intracellular reduced glutathione (GSH), an efficient thiol antioxidant system in the lung, provides protection against oxidants. GSH may be crucial for oxidant-induced NF-κB response [18]. At present, the only antioxidant widely available for patients with COPD is N-acetyl-L-cyteine (NAC) [19,20] which exhibits direct and indirect antioxidant properties and protect cells from oxidative damage [21]. Its free thiol group is capable of interacting with the electrophilic groups of ROS (direct effect), and as a precursor of GSH (indirect effect) increases intracellular GSH level and hence protects the cells against oxidative stress [22,23].

TLR4 signaling is important in lung diseases [24,25]. TLR4 in the lungs could be activated either by conserved microbial component or exogenous oxidants [26] and therefore modulate inflammatory responses. Moreover, there is a link between ROS and TLR4 [18,26,27]. Very recently, we documented that TLR4 mediates CS-induced IL-8 production in monocyte-derived macrophages (MDMs) [8]. Since CS is a rich source of radicals and can induce oxidative stress, we hypothesized that CS-induced oxidative stress may modulate TLR4 expression and NF-κB activation which leads to the release of IL-8. Therefore, the effects of ROS imposed by CS on TLR4 surface and gene expression, as well as, GSH levels were investigated. Our study shows that CS-induced oxidative stress is involved in modulation of TLR4 mRNA and surface protein expression as well as the cascade of TLR4 signaling pathways and cytokine productions.

## Materials and methods

### Reagents

Reagents were purchased from Sigma-Aldrich except were specified. Monocytes were isolated by RossetSep™ (Stem cell Technology) from buffy coats (Sanquin blood bank) see the below. Cells were incubated in RPMI 1640 (BioWhittaker Cambrex Company, Verriers, Belgium), supplemented with 2 mM N-acetyl-L-alanyl-L-glutamine, 100 U/ml penicillin, 100 μg/ml streptomycin, 2% sodium pyruvate and 20 mM Hepes and 10% heat-inactivated fetal calf serum (FCS) (Invitrogen Life Technolog). The mouse antibody against human IκBα and human IRAK-1 were obtained from Santa Cruz biotechnology (Tebu-bio, Heerhugowaard, The Netherlands).

### Cell culture

For culturing human monocyte-derived macrophages, peripheral blood mononuclear cells (PBMC) were separated by density gradient centrifugation (Pharmacia Biotech, Uppsala, Sweden) of buffy coats obtained from normal blood donors as described before [28,29]. Human blood monocytes were obtained using RosetteSep™ (Stem cell Technologies) according to manufacturer's instructions. Briefly, fresh blood was incubated with RosetteSep™ cocktail at room temperature followed by Ficoll-Paque gradient centrifugation (Life Technologies, Cergy Pontoise, France). The enriched monocytes were collected from the Ficoll:plasma interface and purity was assessed by FACS analysis using a FITC-labeled anti-CD14 mAb (95%). Macrophages were obtained by culturing monocytes for 5 days in medium containing 2.5 ng/ml GM-CSF and 25 ng/ml M-CSF (R&D). TLR4 stably transfected HEK-293 cell line (293-htlr4a) and HEK-293 cells stably transfected with the LacZ reporter gene (293-lacz) were purchased from In vivogen [30]. Cells were culture in medium containing Blasticidin (10 μg/ml), and after 5–7 passages, cells were activated as described below.

### Cigarette smoke medium preparation

CSM was prepared as described before [9]. Briefly, a smoking machine (Teague Enterprises, Davis, CA, USA) was programmed to smoke cigarette according to the federal Trade commission protocol (35 ml puff volume for 2 seconds once per minute). The main and side stream smoke from one cigarette (unfiltered Lucky strike^TD^, tar and nicotine concentration 12 and 0.9 mg respectively) was directed through 5 ml culture medium (RPMI without phenol red). Hereafter, absorbance was measured spectrophotometrically and the media was standardized to a standard curve of CSM concentration against absorbance at 320 nm. The optical density (OD) 4 (100%) is the highest OD at this wavelength which was diluted to OD 0.03 (0.75%) and 0.06 (1.5%) and applied to the cells. Freshly prepared CSM was used in all experiments.

### Cell activation

For measuring IL-8 production by CSM, TLR4 stably transfected HEK293 cells or 293-LacZ HEK-293 were stimulated with CSM (0.06 OD) and LPS (100 ng/ml) for overnight. For modulation of TLR4 receptors via CSM, MDMs were preincubated with anti-TLR4, control antibodies or NAC (1 mM) for 30 min and then stimulated with CSM or LPS (100 ng/ml) as a positive control for 4 h. RNA was extracted and TLR4 and GAPDH gene expression were quantified by real-time PCR. To test the involvement of oxidants in IRAK activation by CSM, MDMs were stimulated with CSM (0.06 OD) in the presence or absence of NAC (10 mM) for 30 min.

For evaluation of ROS production by CSM in MDMs, the cells were incubated with either 10 mM of NAC for 20 min and, then cultured with CSM (OD 0.03 and 0.06 OD) at 37°C for 1 h. The cells were diluted to 10^5^/ml in PBS, and incubated with 10 μM of H2DCFDA for 15 min. After the cells were washed twice with PBS, 10^4^, cells were analyzed by FACScan (Becton Dickinson) to determine their fluorescence intensity.

### IL-8 ELISA

Measurement of IL-8 in culture supernatant was performed by using ELISA kits (BD bioscience), according to the manufacture's instruction.

### FACS analysis

Cells (TLR4 stably transfected HEK293 cells, LACz null cells and MDMs) were treated with CSM (0.03 and 0.06 OD) for 3 h and then washed and incubated on ice for 30 min with a PE-conjugated anti-human TLR4 (clone HTA125) or mouse IgG2a as control isotype (eBioscience). In addition, for the detection of intracellular levels of TLR4, cells were permeabilized with permeabilization buffer (eBioscience) and stained with anti-human TLR4 Ab or relevant isotype. TLR4 expression was assessed on a FACScan flow cytometer (BD Biosciences). The relative TLR4 surface or intracellular levels were quantified by subtracting the mean fluorescent intensity (MFI) from the MFI values of isotype matched control for each sample.

### Real-time quantitative PCR

Total RNA was extracted using High Pure RNA Isolation Kit (Roche Applied Science) according to the manufacture's instruction. Quantity and purity of the extract was measured by nanodrop (Wilmington, DE, USA). Equal amounts of total RNA was reverse transcribed into cDNA using oligo-dt and Superscript III (Invitrogen Corporation). Real-time PCR was performed using SYBR Green PCR Master-Mix (ABGene) for 40 cycles on an ABI Prism 7000 sequence detector (Applied Biosystems) according to manufacture's instruction. Amplification was achieved using an initial cycle of 50°C for 2 min and 95°C for 15 min, followed by 40 cycles of 95°C for 15 s and 60°C for 1 min. Melting curve analyses were performed after the completion of cycling to control the specificity of the PCR products obtained. Primers were designed using the Primer Express (Applied Biosystems) software which is as followed: *tlr4 (GeneBank *Accession NM_138554) forward 5'-CTGCCACATGTCAGGCCTTAT-3'; Reverse 5'-AATGCCCACCTGGAAGACTCT; *tlr2 *(*GeneBank *Accession NM_003264) forward 5'-CATTCCCTCAGGGCTCACAG-3'; Reverse 5'-TTGTTGGACAGGTCAAGGCTT-3'; and *gapdh *(*GeneBank *Accession AY340484) forward 5'-CCAGGTGGTCTCCTCTGACTTC-3'; Reverse 5'-CACCCTGTTGCTGTAGCCAAA-3'. The raw Cts (threshold cycle) values from the reactions were analyzed with a modified delta-Ct method with efficiency correction using a PCR data analysis program, qBase to obtain relative quantification values.

### Protein Assay

The protein content of the lyzate was determined using the bicinchonic acid (BCA) kit (Pierce, Erembodegem-Aalst, Belgium). Protein standards were obtained by diluting a stock solution of Bovine Serum Albumin (BSA) (Pierce).

### Western blotting assay

Treated cells were washed once with cold PBS and lysed on ice-cold lysis buffer containing 50 mM Tris (PH 8.0), 110 mM NaCl, 5 mM EDTA, 1% Triton X-100, and PMSF (100 μg/ml) and aprotinin (2 μg/ml). Protein concentration was measured by BCA protein assay kit. Whole cell lysates were boiled and separated on polyacrylamide gel (12%), transferred onto nitrocellulose membrane (Novex). For immunoblotting, membranes were soaked in super-blocking buffer (Pierce) for 1 hour to block" the nonspecific binding of proteins. The nitrocellulose was then incubated with the specific antibody, human IκB-α and IRAK, at appropriate dilutions. Membranes were then washed several times in washing buffer (phosphate buffered saline with 0.05% Tween-20) and incubated with secondary antibody coupled to peroxidase at a 1:10,000 dilution for 1 h. Blots were washed with TBS-T and immunoreactive signals were visualized by an enhanced chemiluminescence reagent (ECL; Amersham). Films were scanned and analyzed on a GS7–10 Calibrated Imaging Densitometer equipped with Quantity One v.4.0.3 software (Bio-Rad).

### Intracellular oxidative activity assay

After stimulation of MDM (10^4 ^cells were washed twice with PBS, and and then intracellular ROS generation was evaluated with a fluorogenic substrate, 2'. 7'-dichlorofluoresceindiacetate (H2DCFDA, Invitrogen). This probe is a non-fluorescent compound which readily diffuses to the cells and becomes fluorescent when oxidized by hydrogen peroxide, peroxinitrite (ONOO-), and hydroxyl radicals (OH^•^). Thus, dye oxidation is an indirect measure of the presence of the reactive oxygen intermediate/species, calculated by dividing the mean channel fluorescence of a treated sample by that of the untreated one and multiplying by 100 to obtain the relative change, expressed as a percentage.

### Measurement of cellular GSH content

Intracellular GSH content was assessed in cellular lysate according to the methods of Tietze [31] with slight modification [32]. Briefly, washed cells were lysed by repeatedly freezing and thawing using lysis buffer containing 0.6% (w/v) sulfosalicylic acid. 0.1% (v/v) Triton X-100, 5 mM EDTA in 0.1 M potassium phosphate buffer, PH 7.5. The supernatant collected after centrifugation and incubated with 0.2 mg/ml dithiobisnitrobenzoic acid (DTNB) and 1.67 U/ml glutathione reductase in phosphate buffer-EDTA for 30 seconds, then 0.2 mg/ml β NADPH was added and the rate of DTNB reduction was spectrophotometrically measured at 405 nm. GSH content was calculated using a standard curve, and expressed as nmol/mg protein.

### Data analysis

Data are presented as means ± SEM. Comparison between groups was performed by using un-paired *t *tests. A *P *value of less than 0.05 was taken as statistically significant.

## Results

### TLR4 is involved in CSM-induced IL-8 production

**Figure 1:**
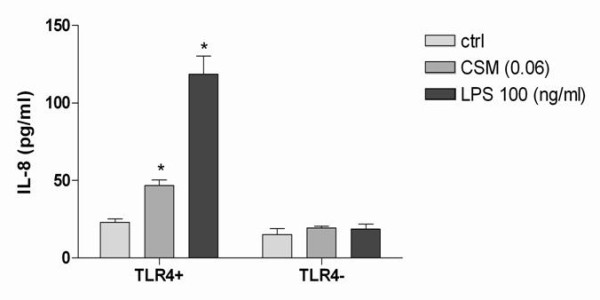
**TLR4 involved in CSM-induced IL-8 production**. TLR4 stably transfected HEK293 cells or 293-LacZ HEK-293 cells (2 × 10^6^/ml) were stimulated with CSM (0.06 OD) and LPS (100 ng/ml) for overnight. Supernatant were analyzed for IL-8 production by ELISA. Assays were performed in duplicate a minimum of three times. Values are expressed as mean +/- S.E. (n = 3). * signifies (**P = 0.01) of observed effect vs. control.

Recently, we demonstrated that CSM-induced IL-8 production by MDMs could be inhibited by neutralizing antibodies against TLR4 [8]. To support these effects of CSM in detail, we investigated in TLR4 stably transfected and null HEK 293 cell lines. TLR4 stably transfected HEK 293 cells were stimulated with CSM (0.06 OD) or LPS (100 ng/ml) as a positive control. As depicted in Fig. 1 CSM and LPS induced IL-8 release only in TLR4 stably transfected HEK293 cells but not in LacZ HEK 293 cell line.

### CSM modulates expression of TLR4

**Figure 2:**
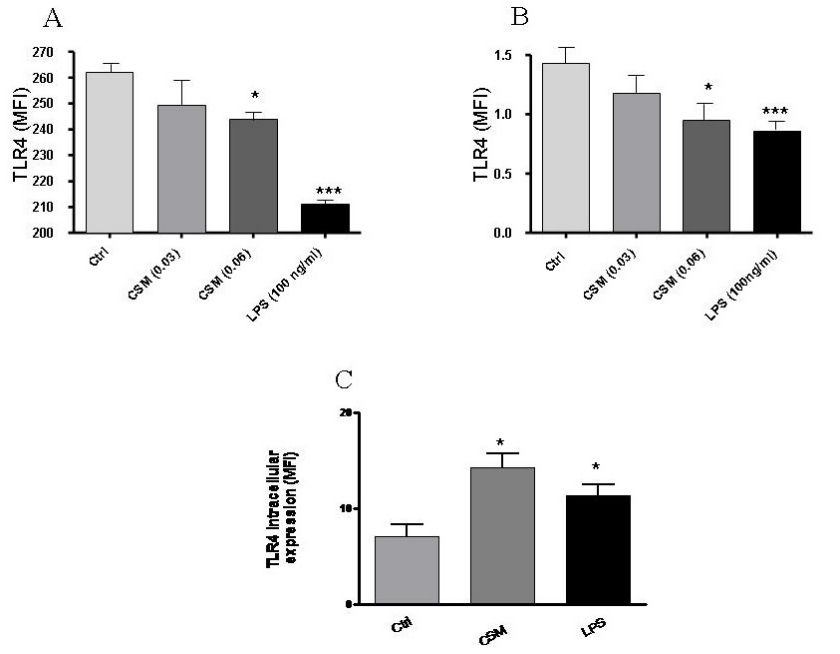
**Modulation of TLR4 expression by CSM**. TLR4 stably transfected HEK293 cell (A) or MDMs (B) were treated with CSM (0.03 and 0.06 OD) for 3 h and then incubated with PE conjugated anti-TLR4 or isotype control antibody as described in materials and methods. FACS analysis of a representative of at least 3 experiments showing the mean fluorescence intensity (MFI) difference of each group. Values are expressed as mean +/- S.E.M (n = 3). *p = 0.05,***p = 0.001 significantly different compared to control. C) MDMs were stimulated with CSM (0.06 OD) or LPS (100 ng/ml) for 3 h and then intracellular levels of TLR4 were measured as described in material and methods. Values are expressed as mean +/- S.E.M (n = 3). *p = 0.05, significantly different compared to control.

In both, MDMs and TLR4 stably transfected HEK 293 cells, CSM induced a concentration-dependent decrease in surface expression of TLR4 (Fig. 2A and 2B). LPS as a positive control induced a more pronounced decline in TLR4 surface expressions in HEK293 cells than in MDMs.

Next, we investigated whether the surface suppression of TLR4 was due to the internalization/shedding of receptors. Therefore, intracellular level of TLR4 expression was studied. As shown in Fig. 2C, CSM at the same time points, intracellular levels of TLR4 in MDM was increased.

**Figure 3:**
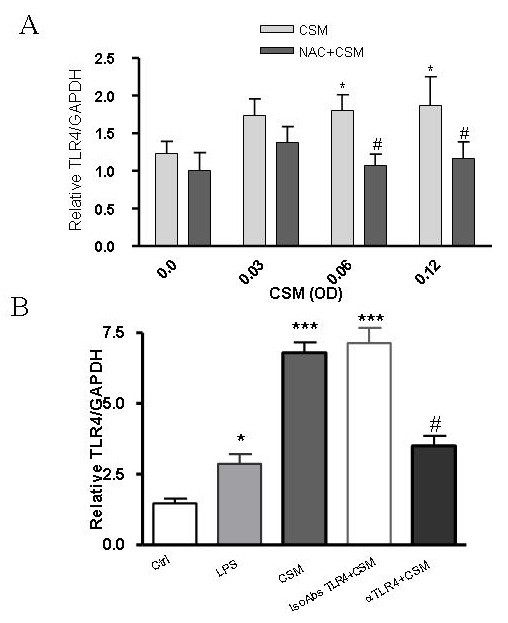
**CSM regulates expression of TLR4 via ROS**. (A) MDMs (5 × 10^6 ^cells) were stimulated by CSM (0.03, 0.06 and 0.12 OD) for 4 h with and without pretreatment with NAC (10 mM) for 30 min. RNA was extracted and TLR4 and GAPDH gene expression were quantified by real-time PCR. Results are expressed as copies of TLR4 vs. copies of GAPDH gene. (B) MDMs were preincubated with naturalizing anti-TLR4 or isotype control antibodies for 30 min and then stimulated with CSM (0.06 OD) for 4 h or LPS (100 ng/ml) and mRNA levels of TLR4 was determined by real-time PCR method. Values are expressed as mean +/- S.E.M (n = 3).*P < 0.05,***p = 0.001 significantly different compared to control and # P < 0.05 significantly different compared to CSM stimulated (n = 3).

To further study the effects of CSM on modulation of TLR4 expressions, mRNA levels of TLR4 was studied by using PCR. MDMs were incubated with CSM (0.03, 0.06 and 0.12 OD) for 4 h and RT-PCR was performed by using the human TLR4 and GAPDH primers as a reference gene. CSM upregulated the expression of mRNATLR4 in MDMs (Fig. 3A) and pre-incubation with NAC suppressed this effect. Pre-incubation of MDMs with a neutralizing antibody against TLR4 (20 μg/ml) decreased the mRNA levels of TLR4 enhancement to CSM (about 50%) while no inhibition was observed when cells were pre-incubated with the control antibody (Fig. 3B). Similar to CSM, LPS as a positive control enhanced the TLR4 mRNA expression.

**Figure 4:**
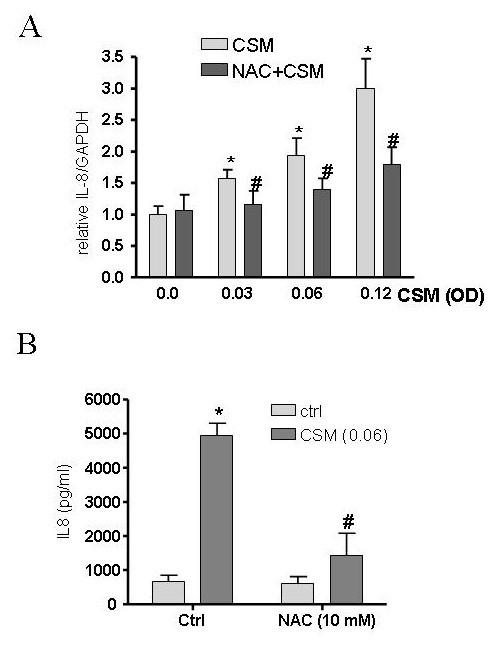
**IL-8 expression is ROS dependent after CSM exposure**. MDMs (5 × 10^6 ^cells/ml) were pretreated with NAC (10 mM) for 30 min and then stimulated by CSM (0.03, 0.06 and 0.12 OD) for 4 h. RNA was extracted and mRNA levels of IL-8 were quantified by real-time PCR (A). Results are expressed as copies of IL-8 vs. copies of GAPDH mRNA. (B) MDMs (1 × 10^6 ^cells/ml) were pretreated with NAC (10 mM) for 30 min and then stimulated by CSM (0.06 OD) for 16 h Supernatants were collected after 16 h incubation and IL-8 production was quantified using ELISA methods. *P < 0.05 vs baseline # P < 0.05 vs CSM stimulated (n = 3).

Next, in order to investigate the involvement of ROS by CSM, MDMs were pre-treated with the antioxidant NAC (10 mM) for 30 min and then incubated with CSM (0.03, 0.06 and 0.12 OD) for 4 h. NAC suppressed the upregulation of TLR4 mRNA-induced by CSM compared to control (Fig. 3A). Moreover, NAC suppressed the expression of IL-8 at mRNA and protein levels (Fig. 4A and 4B).

### CSM induces the generation of ROS by MDMs

**Figure 5:**
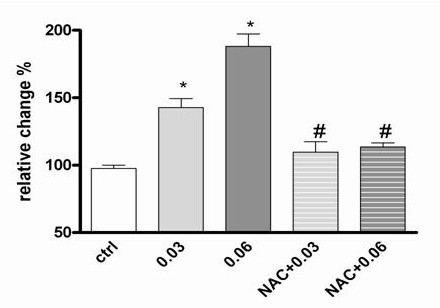
**CSM induces generation of ROS in MDMs**. MDMs were pretreated with NAC (10 mM) for 30 min and then stimulated with CSM (0.03 and 0.06 OD) for 1 h. Intracellular ROS concentration was measured by incubation of cells with H2DCFDA as a probe for 30 min at 37 *o*C. Then after washing, the density of flurochrom as indicator for generation of ROS was determined by FACS analysis. The results were expressed as fold increase over control cells. Data represent means ± SEM of triplicate experiments (n = 3). * p < 0.05 versus unstimulated control; # p < 0.05 versus CSM.

Further, we directly measured ROS production by using a fluorescence probe (H2DCFDA). As demonstrated in Fig. 5, exposure of MDMs to CSM (0.03 and 0.06 OD) induces a dose-dependent oxidation of the fluorescence probe which indicates intracellular ROS production by CSM (oxidative activity). ROS production by CSM was completely blocked when the cells were pre-incubated with NAC (10 mM).

### ROS generation by CSM, enhanced phosphorylation of IRAK and induces IκB-α degradation

**Figure 6:**
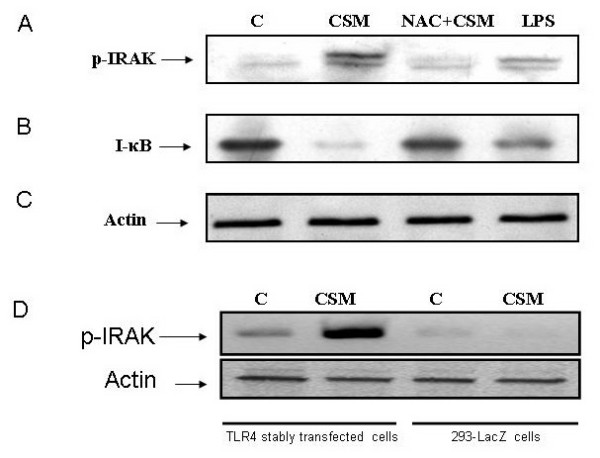
**CSM regulates phosphorylation of IRAK and degradation of IκB-α by MDMs and phosphorylation of IRAK in HEK cells**. MDMs (3 × 10^6 ^cells) were pretreated with NAC (10 mM) for 30 min and then stimulated with CSM (0.06 OD) and LPS (100 ng/ml) for 30 min as described at material and methods section. The expression of phospho IRAK (A) and IκB-α degradation (B) were determined by whole lysates of cells by Western blot analysis. Representative results of three independent experiments and β-actin (C) served as loading controls from cytoplasm. D) TLR4 stably transfected HEK293 cells or 293-LacZ HEK-293 cells (3 × 10^6 ^cells) were stimulated with CSM for 30 min as described at material and methods section. The expression of phospho IRAK were determined by whole lysates of cells by Western blot analysis. Representative results of three independent experiments and β-actin served as loading controls from cytoplasm.

It has been show that that IRAK phosphorylation is the first step after MyD88 recruitment which finally leads to degradation of the IκB-α and activation of NF-κB [8]. Stimulation of the MDMs with CSM for 30 min induced the phosphorylation of IRAK which was abolished by adding NAC (Fig. 6A). Moreover, CSM and LPS (as a control) degradated IκB-α and preincubation of MDMs with NAC suppressed the degradation of IκB-α induced by CSM (Fig. 6D).

Next, to confirm specific effects of CSM on TLR4 signaling, the phosphorylation of IRAK in TLR4 stably transfected HEK cells and null cells were studied. CSM induced phosphorylation of IRAK in TLR4 stably transfected HEK cells but not in null cells (Fig. 6C).

### CSM modulates GSH levels

**Figure 7:**
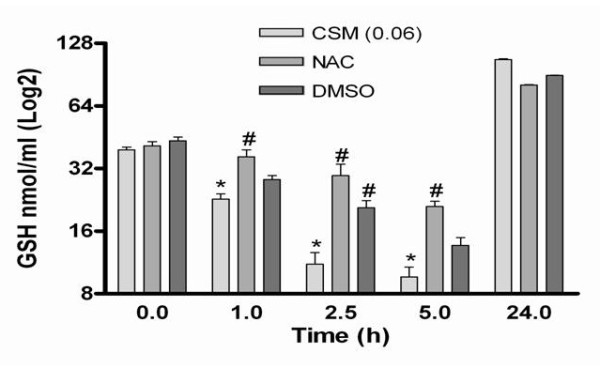
**Antioxidant prevents intracellular GSH depletion-induced by CSM**. MDMs (5 × 10^6 ^cells) were pretreated with NAC (10 mM) and DMSO (2%) for 30 min and then stimulated with CSM (0.06 OD) at various time points (1, 2.5, 5, and 24 h). Intracellular GSH contents were measured by cellular lysate as described at "material and methods" section and expressed as mean ± SEM of medium-treated cells. * p < 0.05 versus un-stimulated control; # p < 0.05 versus.

We measured the levels of GSH in MDMs after CSM stimulation at various time points. CSM time-dependently decreased GSH concentrations for 5 h and after long time exposure this effects was restored (Fig. 7). Preincubation of cells with NAC (10 mM) and DMSO (2%) for 20 minutes restored/attenuated the loss of intracellular GSH levels at all time points. The period and concentration of NAC and DMSO was chosen on the basis of previous studies with these agents [18,33].

## Discussion

TLRs are found on the cell surface and in endosomes of many different cell types. To date, 13 TLRs have been identified in mice and humans with corresponding synthetic or naturally occurring ligands. One of them is TLR4 which recognizes lipopolysaccharides (LPS) from gram negative bacteria [13].

We have demonstrated earlier that CSM induces IL-8 production via TLR4 in MDM. Interestingly; this effect was not due to contamination of LPS [8]. In the current study these pervious observations were extended in more details.

First, as supportive evidence, we employed the HEK293 cells as stably transfected TLR4 and LACz HEK293 as a control cell lines. Only in TLR4 stably transfected HEK cells, CSM induced the production of IL-8. Moreover, CSM regulates surface and intracellular TLR4 expression in MDMs.

Interestingly, CSM induced the internalization of TLR4 receptor. TLR4, in the lung, not only could recognize microbial components but also could sense either exogenous oxidants like electrophilic compounds and free radicals present in CSM or endogenous oxidants [34-36]. Activation of TLRs can lead to inflammatory response by signaling through NF-κB, the best characterized regulator of TLR signaling [16]. Cigarette smoke is a source of potent reactive oxygen and nitrogen species which participate in intracellular signaling and NF-κB activation [8]. In addition, several studies have revealed the importance of oxidative stress in the IL-8 productions [37,38]. Thus, we studied the role of ROS on CSM-induced increase in mRNA TLR4 activation of MDMs. It was found that NAC abrogated the expression of TLR4 expression. Furthermore, NAC interfered with CSM-induced IL-8 production through a mechanism that is associated with increased ROS production and GSH depletion.

GSH levels decreased dose- and time- dependently and pre-treatment of the cells with antioxidants NAC and DMSO prevented the CSM-induced decrease in GSH levels in MDMs. Since NAC is able to scavenge a wide range of oxidants (hypochlorous acid, hydrogen peroxide, superoxide and hydroxyl radical) it revealed a better antioxidant effect compare to DMSO which reacts with the hydroxyl radical [22]. By using a direct approach to measure ROS production, CSM dose dependently increases intracellular ROS generation by MDMs. These findings may suggest that CSM induces its effect by intracellular ROS generation and direct electrophilic ability to decrease intracellular GSH.

Despite of the decreased surface expression of TLR4 after CSM, a delayed up-regulation might be induced by a protective mechanism like the enhancement of GSH. Surface attenuation of TLR4 receptor may be explained by an internalization/shedding of the receptor complex or by changes in the structure of the receptor to cross-link with other TLR4 molecule since recent evidence indicates that cross-linking is necessary for signal transduction [39]. Cross-linking of receptors or receptor clustering by thiol-reactive mercury or ultraviolet radiation have been documented which activates downstream signaling [40,41]. The downregulation of TLR4 receptor presented here is in contrast to the result from experiments with RAW 264.7 cells exposed to hydrogen peroxide (H2O2)[34]. It is not clear whether this discrepancy reflects genetic differences between human and mice [42], cell differences or the type of oxidant.

Next, the TLR4 expression at mRNA levels was studied. We and found that CSM increases mRNA levels of TLR4. Upregulation of mRNA level inside cells could lead to upregulation of intracellular protein levels of TLR4 which is reflected by increased intracellular expression.

The antioxidant NAC prevented the upregulation of TLR4 mRNA which indicates a role of oxidative stress induced by CSM. NAC prevents the oxidative stress via counteracting with electrophilic group of ROS (direct effect) or stimulating the synthesis of the cellular GSH levels and therefore protecting the cells against oxidants (indirect effect) by modulating the redox signaling pathways [22,23]. Thus these results indicate that CSM by inducing ROS generation, may modulates the expression of TLR4.

TLRs ligations lead to recruitment of many proteins to the cytoplasmic domain of the receptor like adapter molecules MyD88. MyD88 recruits and promotes the interaction between IL-1R-associated kinases (IRAK)-4 and IRAK-1, resulting in the phosphorylation and activation of IRAK-1 by IRAK-4 [43,44]. Subsequently, dissociation of IRAK1 from the receptor complex and association with the signal transducer tumor necrosis factor receptor-associated factor 6 (TRAF6) occur. The subsequent downstream signaling leads to the degradation of the IκB-α and activation of NF-κB [45-47]. CSM induced the phosphorylation of IRAK-1 and degradates IκB-α [8]. In this study by using NAC, we have demonstrated that ROS play an important role in CSM-induced TLR4 associated intracellular signaling. Interestingly, we have found that CSM specifically induced phosphorylation of IRAK-1 in stably transfected TLR4 HEK cells but not in null TLR4 cells.

In conclusion, these results indicate that CSM induces a ROS mediated signal transduction pathway via TLR4 in MDMs. Induction of oxidative stress plays an important role in the regulation of TLR4 and the production of IL-8.

## Abbreviations

COPD: Chronic Obstructive Pulmonary Disease; TLR4: Toll-like receptor-4; ROS: reactive oxygen Species; CSM: Cigarette Smoke Medium; CS: Cigarette smoke; IL-8: interleukin-8; NAC: N-acetyl-L-cysteine; OD: Optical Density; TNF-α: Tumor necrosis factor-α; GSH: Glutathione; CS: Cigarette smoke; MDMs: monocyte-derived macrophages; LPS: Lipopolysaccharide.

## Competing interests

The authors declare that they have no competing interests.

## Authors' contributions

HS and EM equally conceived of the study, and participated in the design of the study and performed immunoassays, FACS analysis, statistical analysis, and wrote the first draft and final version of the manuscript. KK, AK IR and FN participated in designing the experiments and took part in critical revision of the manuscript. FN participated in the design and coordination of the study. GF conceived of the study, and participated in the design of the study and supervised the project. All authors read and approved the final manuscript.
